# Fostering Psychophysical Well-Being via Remote Self-Managed Empowerment Protocols: A Scoping Review

**DOI:** 10.3390/brainsci15111194

**Published:** 2025-11-05

**Authors:** Davide Crivelli, Benedetta Vignati

**Affiliations:** 1 International Research Center for Cognitive Applied Neuroscience (IrcCAN), Università Cattolica del Sacro Cuore, 20123 Milan, Italy; 2 Faculty of Psychology, Università Cattolica del Sacro Cuore, 20123 Milan, Italy; benedetta.vignati1@unicatt.it

**Keywords:** neuroempowerment, self-managed intervention, bio/neuro-feedback, mindfulness, embodied awareness, wearables, well-being, participant agency, remote training

## Abstract

Remote, self-managed neuroempowerment protocols are emerging as promising tools for promoting psychophysical well-being in healthy individuals through scalable, home-based interventions. Rooted in positive psychology, applied psychophysiology, and embodied awareness practices, these protocols increasingly leverage wearable technologies and digital platforms to support self-regulated training in cognitive, emotional, and physical domains. This scoping review explores the current literature on such interventions, guided by a triadic model of subjective well-being encompassing neurocognitive efficiency, psychological balance, and physical fitness. A systematic search across major scientific databases identified 28 studies meeting inclusion criteria, with a focus on home-based interventions targeting healthy adult populations using embodied awareness practices, applied psychophysiology techniques, and empowerment-based strategies. Findings indicate that these interventions yield improvements in attention regulation, stress reduction, and subjective well-being, particularly when combining digital feedback systems with embodied practices. However, significant methodological limitations persist, including the overreliance on self-report measures, lack of longitudinal follow-up, and insufficient integration of objective, multimodal assessment tools. Moreover, few studies explicitly address the role of participant engagement and agency—key elements in neuroempowerment frameworks that conceptualize the individual not as a passive recipient of treatment, but as an active agent in the training process. This review highlights the need for more rigorous and theoretically grounded research, advocating for integrative, adaptive intervention models supported by wearable neurotechnologies. Such approaches hold the potential to enhance motivation, personalize feedback, and promote sustainable well-being in ecologically valid, participant-centred ways.

## 1. Introduction

Home-based interventions have long played a role in psychological practice, and the progress of the web-based utilities and widespread use of digital technologies—such as smartphones and tablets—has dramatically expanded their accessibility and implementation [1,2,3,4]. These interventions are typically categorized under the broader paradigms of tele-health and e-Health. The term e-Health denotes the use of information and communication technologies (ICTs) to enhance the prevention, diagnosis, monitoring, and treatment of mental and physical health. A subset of this approach, mHealth, refers specifically to mobile applications that support health-related behaviours and interventions.

Remote neuroempowerment protocols have recently gained considerable attention for their ability to deliver accessible, technology-enhanced interventions aimed at optimizing mental well-being and cognitive functions in healthy individuals [3,5,6,7,8]. These protocols are often characterized by their integration of cognitive training and/or embodied awareness techniques with wearable sensing systems, which allow for real-time monitoring, feedback, and adaptive adjustment based on users’ physiological and cognitive states. Embodied awareness interventions, which focus on enhancing bodily consciousness and self-regulation, represent a promising frontier for preventive health and resilience training in non-clinical populations [9,10,11,12]. The convergence of behavioural science, neuroscience, and wearable technology positions remote neuroempowerment protocols as scalable and user-centric tools for fostering sustainable mental wellness.

Despite increasing interest and technological capability, much of the existing research on remote and digital interventions has focused predominantly on clinical applications, e.g., [13,14,15,16,17]. This ‘context bias’ has led to a relative lack of evidence guiding the development of proactive, self-managed interventions for healthy populations. Thus, there remains a pressing need to explore how remote neuroempowerment protocols can support subjective well-being through everyday practice and self-monitoring.

This review proposes a comprehensive assessment of the potential for remote, self-managed empowerment protocols to promote well-being in healthy individuals. Specifically, it draws on an integrated model of subjective well-being grounded in the interdependence of three foundational pillars: neurocognitive efficiency, physical fitness, and psychological balance. This triadic framework underscores how these domains function not in isolation, but in a dynamic interplay that sustains a high quality of life.

### 1.1. Theoretical Background: The Three Pillars of Well-Being

Subjective well-being is best understood as the result of dynamic and reciprocal interactions among many components. In this review, we use a triadic model of subjective well-being as the theoretical frame of reference, where psychological balance, neurocognitive efficiency, and physical fitness form a conceptual triad whose synergistic integration sustains and enhances an individual’s quality of life. Rather than acting in isolation, each domain modulates and is modulated by the others, creating a feedback loop that underlies adaptive functioning across cognitive, physical, and emotional spheres.

Psychological balance refers to an individual’s capacity to maintain emotional stability, self-acceptance, and adaptive responses to life challenges. Drawing on frameworks such as Ryff’s psychological well-being model [18], this domain encompasses dimensions such as autonomy, personal growth, and life purpose. Trait variables like optimism and resilience, alongside state-dependent regulatory capacities, underpin psychological balance. Notably, this domain also mediates the integration of cognitive and physical functioning: individuals with greater psychological balance are better able to derive benefit from physical activity and cognitive training, owing to enhanced emotion regulation and motivational resources [19,20].

Neurocognitive efficiency encompasses the capacity for rapid, flexible, and resource-efficient cognitive processing, particularly in domains such as executive control, attention orienting, working memory, and self-regulation. Empirical studies have demonstrated that individuals with high neurocognitive efficiency exhibit greater resilience to psychological and physical stressors and enhanced adaptability across contexts [21,22]. This domain is an object of growing attention both as a target of assessment and a target for enhancement using psychophysiological methods and neurofeedback paradigms, which allow for fine-grained investigation and targeted intervention. Programs aimed at enhancing neurocognitive efficiency—including embodied awareness protocols, cognitive training, and adaptive neurofeedback—have been shown to foster not only mental flexibility but also greater self-regulatory capacity and neurocognitive efficiency [8,9,23,24,25,26,27,28].

Physical fitness, traditionally associated with cardiovascular health and muscular strength, also exerts profound effects on neural functioning and psychological resilience. Evidence from longitudinal and intervention studies links regular physical activity—especially aerobic activity—with improvements in executive functioning, mood regulation, and stress resilience [29,30,31]. From the biological perspective, it has been suggested that these effects could be mediated, among other phenomena, by increased neurotrophic factors such as BDNF, improved neurovascular coupling, and reduced systemic inflammation [29]. Furthermore, we posit that physical fitness could be a critical enabling factor for increasing and sustaining individuals’ opportunities and capacity to engage with and benefit from cognitively or emotionally demanding interventions over time, reinforcing its role as a foundational pillar of well-being.

The synergy among these pillars justifies multi-domain interventions that combine cognitive training, physical exercise, and embodied awareness practices. In keeping with the threefold model, neurocognitive efficiency may sustain the efficacy with which physical exertion is managed, while physiological vitality enhances cognitive endurance and emotional resilience. Finally, psychological balance provides the motivational scaffolding necessary for sustained behavioural change across both cognitive and physical domains.

Among the most promising strategies for fostering the three pillars are meditation-based interventions and neurofeedback training. Meditation practices—especially mindfulness-based ones—have been shown to reduce stress, improve emotion regulation, and enhance attention—effects that directly support both neurocognitive efficiency and psychological balance [10,32,33,34,35]. Recent evidence suggests that the integration of non-invasive neurofeedback can further amplify these benefits by providing real-time biofeedback, thereby enabling users to develop self-regulation strategies based on their own physiological data [8,24,36]. Wearable technologies extend the potential of these practices by allowing continuous monitoring and feedback outside the lab or clinical setting. Such tools facilitate the transition of well-being practices from controlled environments to daily life, empowering users to independently manage and optimize their own psychophysiological states.

Thus, the triadic model presented here offers a robust theoretical foundation for designing remote neuroempowerment protocols that are ecologically valid, scalable, and adaptive. Through wearable sensor technology and digitally mediated feedback systems, individuals can engage with interventions that simultaneously target cognitive, emotional, and physical domains—thereby supporting sustainable and personalized trajectories of well-being enhancement.

### 1.2. Aims of the Review

This scoping review aims to summarise the available scientific literature on the efficacy of remote, self-managed empowerment protocols in sustaining and enhancing psychophysical well-being in healthy populations, with a specific focus on psychological balance and neurocognitive efficiency. It emphasizes intervention methods grounded in applied psychophysiology and embodied awareness practices, including mindfulness and neurofeedback. Also, a secondary purpose of this work is to begin outlining a knowledge base to inform implications for practice and research on remote neuroempowerment interventions targeting the well-being of healthy populations in the lifespan.

Two specific questions guided this review. The first question was focused on the effectiveness of home-based self-managed empowerment protocols in improving psychological balance and neurocognitive efficiency of healthy individuals. The second question concerned the diffusion of wearable neurotechnologies as supportive devices in home-based self-managed empowerment protocols devoted to fostering psychophysical well-being in healthy people.

## 2. Methods

The literature survey of this scoping review was conducted in accordance with the PRISMA Extension for Scoping Reviews standards as reporting guidelines [37].

### 2.1. Search Strategy

A comprehensive keyword search was conducted in four electronic databases: Medline, APA PsycINFO, Scopus, and PubMed, to identify studies meeting the eligibility criteria. Searches were performed between 1 June and 30 September 2024.

Search terms combined index terms and text words relating to web-based interventions, neurofeedback, well-being, healthy conditions, and study design. Search terms included the following:

(eHealth OR “web-based” OR online OR “home-based” OR computer OR internet OR mobile OR eTherap* OR “smartphone app” OR “web app” OR “computer-based”) AND (training OR intervention OR treatment OR empowerment OR enhancement) AND (wellbeing OR “well-being” OR “cognitive function”) AND (mindfulness OR “mind-fulness” OR meditation OR yoga OR “mindful yoga” OR neurofeedback OR “embodied awareness” OR wearable) NOT (patholog* OR clinical OR illness OR disorder).

Reference lists of included papers and relevant reviews were also screened to identify additional eligible studies.

### 2.2. Eligibility Criteria

Studies were included in the systematic review if they met these following criteria: (a) targeted at individuals aged ≥ 18 years; (b) focused on cohorts of healthy subjects; (c) implemented a structured training program grounded in applied psychophysiology, em-bodied awareness, or empowerment-based techniques targeting well-being and positive psychology themes; (d) included at least one home-based or remotely delivered intervention component supported by digital or wearable technology; (e) were published after January 2015 in peer-reviewed journals.

Studies were excluded if they (a) were reviews, meta-analyses, books, conference papers, or other secondary sources; (b) did not investigate outcomes related to neurocognitive efficiency or psychological balance; (c) focused on digital psychotherapy or purely clinical treatments; (d) used gamified platforms as the main intervention vehicle; or (e) involved extremely brief protocols (duration < 1 week).

### 2.3. Selection Process and Data Extraction

Two independent reviewers (D.C. and B.V.) conducted the literature search, screening, and selection of studies. Titles and abstracts were first reviewed for relevance; full-text articles were then assessed for eligibility according to the criteria above. For each included study, data were extracted using a standardized form that included (i) sample characteristics; (ii) study design; (iii) intervention type and duration; (iv) main outcome measures; and (v) principal results. These extracted elements are summarized, together with other data, in Table 1.

Disagreements between reviewers during screening or extraction were discussed collaboratively with reference to the predefined inclusion and exclusion criteria. When consensus could not be reached, a third independent judge with expertise on the topics of investigation was consulted to ensure neutrality and consistency.

### 2.4. Integration with PRISMA Flow Diagram

**Figure 1:**
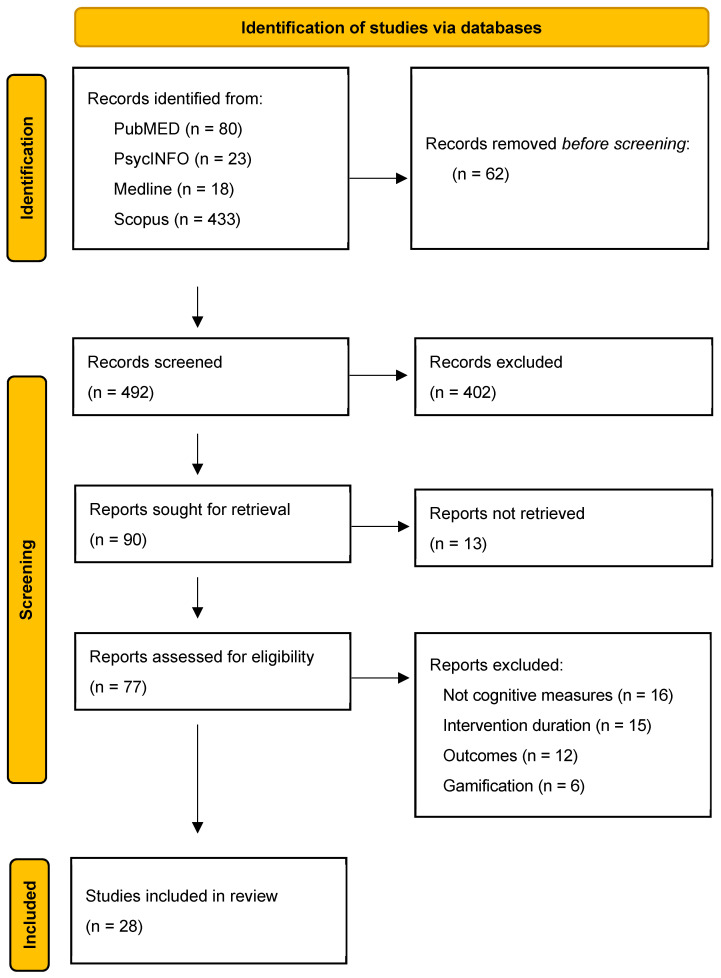
PRISMA 2020 flow diagram for the scoping reviews, summarizing the outcome of identification, screening, and inclusion steps.

The number of records identified, screened, excluded, and included is reported in Figure 1 (PRISMA 2020 flow diagram). The exclusion reasons described in the text correspond to those represented in the diagram: (i) lack of cognitive or psychological outcome measures; (ii) insufficient intervention duration; (iii) irrelevant outcome domains; or (iv) inaccessibility of full text. Please refer to Section 3 for a complete report on the outcome of the selection process and on excluded studies.

## 3. Results

We conducted a comprehensive review of studies retrieved from scientific databases, encompassing a wide range of experimental settings. The literature includes numerous articles addressing remote interventions, a topic that has seen substantial growth following the COVID-19 pandemic.

A significant portion of these publications focuses on the efficacy and validity of online psychotherapy, e.g., [64,65,66]. However, these studies were excluded from our analysis as they did not meet the inclusion criteria, primarily due to the absence of reinforcement-based training components or a specific focus on neuroempowerment. Additionally, many articles examined remote treatment and prevention protocols administered to clinical populations, such as individuals with cancer, multiple sclerosis, or other non-communicable diseases [15,64,67,68]. These, too, were excluded on the basis of population or intervention characteristics that fell outside our predefined parameters.

The remote interventions included in this scoping review are heterogeneous, targeting diverse populations and utilizing a variety of modalities. Several studies relied on online meeting platforms such as Zoom, while others focused on smartphone-based applications (see Table 1). A subset of studies explored the use of wearable technologies, including biofeedback and neurofeedback devices (see Table 1).

From an initial pool of over 550 articles, a substantial number were excluded due to not meeting the inclusion criteria or due to inaccessibility of the full text (see PRISMA flow diagram, Figure 1). Specifically, 62 articles were excluded prior to screening due to duplication or failure to meet basic eligibility (e.g., not in English). After title and abstract screening, 402 articles were excluded following abstract review due to specific exclusion criteria such as (i) study focusing on clinical or pathological populations (e.g., individuals with chronic illness or mental disorders); (ii) intervention lacking a self-managed, home-based components; (iii) study addressing psychotherapy platforms without empowerment or training components; (iv) intervention using gamified or entertainment-based platforms. Then, of the 90 full-text articles finally sought for retrieval, 13 could not be obtained due to access limitations.

Reports assessed for eligibility were 77 and, of these, 49 were excluded for the following reasons: 16 studies did not include outcome measures related to neurocognitive efficiency or psychological balance; 15 studies involved interventions that were too brief to be considered beyond acute exposure to specific intervention techniques; 12 studies did not target subjective well-being as a primary outcome; and 6 studies employed gamified intervention formats.

Ultimately, 28 studies met all inclusion criteria and were selected for detailed analysis. A summary of the selection process is shown in the PRISMA 2020 flow diagram (Figure 1), and the included studies are described in Table 1.

### Study Characteristic

The 28 studies included in this review were published between 2015 and 2024. A detailed summary of the study selection is provided in Table 1.

As shown in the table, a wide range of methodologies and intervention targets were employed across the studies. All included articles are grounded in the theoretical frameworks of positive psychology and the psychology of well-being, with a particular emphasis on empowerment-oriented interventions.

Although all participants were healthy individuals across the lifespan, a key distinction among the selected studies lies in the characteristics of their sample populations. The 28 studies encompassed a broad spectrum of groups, including meditation practitioners [57], healthy adults [23,24,43,46,51,54,55,56,60,63], athletes [39,62], employees [44,50,58,61], healthcare professionals [49], law enforcement officers [45], and students [40,41,42,47,48,53,59].

Many interventions combined principles of positive psychology with remote mindfulness training, yielding favourable outcomes. A substantial number of studies utilized web-based or mobile application platforms [40,41,42,44,46,47,50,51,54,55,56,57,58,59,60,62]. Other studies adopted home-based protocols incorporating wearable neurofeedback devices to enhance training efficacy [23,24,38,39,43,61,63].

Across the reviewed literature, improvements were commonly observed in both psychological and cognitive outcomes. These findings support the notion that a multimodal approach—assessing both psychological and cognitive dimensions—may offer a more comprehensive understanding of individual empowerment processes.

## 4. Discussion

This scoping review has mapped a body of literature that collectively highlights the transformative potential of remote, self-managed neuroempowerment protocols for enhancing psychophysical well-being in healthy individuals. These interventions—grounded in applied psychophysiology, embodied awareness practices, and the use of digital and wearable neurotechnologies, yet self-managed—seem to be able to support the development of psychological balance, neurocognitive efficiency, and—with a few examples—physical fitness, which together form a synergistic triad sustaining subjective well-being.

While the reviewed literature demonstrates promising outcomes for such forms of remote interventions for well-being, several methodological limitations emerge that constrain the robustness, interpretability, and generalizability of currently available evidence base. Acknowledging these limitations is crucial to guiding future research designs and enhancing the scientific validity of this growing field.

Firstly, a large portion of studies employed pre-/post-intervention designs without long-term follow-ups, thereby limiting the ability to assess changes over time or the durability of intervention effects, with only a few including multiple longitudinal assessment sessions to determine the durability of effects [43,46,47,48,55,57,58,60]. This represents a major limitation, as the core aim of neuroempowerment protocols is to foster sustainable change in psychophysiological and cognitive domains. Without evidence of maintained benefits over time, the long-term efficacy of most protocols remains uncertain.

Another issue concerns the lack of appropriate control conditions. In several studies, the control group was passive—such as a wait-list group [49,50,51,52,53,54,55,56,57,58,59,60,62,63]—or entirely absent [61,62,63]. These methodological shortcomings hinder the ability to rigorously evaluate the efficacy of the tested interventions and the ability to isolate the specific effects of the intervention from general expectancy effects, motivational improvements, or placebo influences, thus limiting the strength of the conclusions that can be drawn. Future studies should prioritize the inclusion of well-defined active controls that match the experimental condition in terms of contact time and structure but lack the specific mechanisms under investigation.

Thirdly, part of the reviewed studies relied on small sample sizes, often around or below 15 participants per condition, especially after considering attrition, drop-outs, or sample reductions due to technical issues in data collection [23,43,61,62]. While such methodological issues may derive—in specific cases—from limitations in the applicability of brain/body-sensing technologies with large samples, accessibility of the target population, or difficulty in reaching and recruiting peculiar populations (e.g., elite athletes, specific professional profiles, …), it limits statistical power and increases the risk of Type I or Type II errors. In addition, demographic imbalances—such as age, gender, and cultural background—were often underreported or poorly controlled. These constraints reduce external validity and hinder the exploration of individual differences in responsiveness to the interventions.

Fourthly, while self-report tools remain essential for assessing subjective experience and perceived well-being, their widespread use as sole outcome measures introduces significant limitations [40,41,44,45,46,48,49,50,51,52,54,56,57,60,62]. Self-reports are vulnerable to social desirability bias, lack of introspective accuracy, and limited sensitivity to small or latent changes. Critically, in interventions that aim to enhance self-awareness and bodily regulation, the tools used must be able to detect meaningful changes that may initially elude participants’ conscious appraisal. As such, the exclusive reliance on questionnaires can obscure nuanced gains in cognitive flexibility, interoception, or attentional control.

Furthermore, only a few studies adopted a multimethod assessment framework capable of capturing the full spectrum of psychological, cognitive, and physiological change [24,38,39,43,53,58,61]. This is a critical shortcoming, especially in light of a multi-componential model of well-being that often underpins neuroempowerment interventions. A more comprehensive methodological approach would integrate psychometric, neurocognitive, and psychophysiological tools (e.g., EEG, HRV, behavioural tasks), thereby increasing sensitivity to change and enabling a richer understanding of underlying mechanisms.

And again, although the triadic framework introduced in this review includes physical fitness as one of the foundational pillars of subjective well-being, the majority of the studies identified focused solely on psychological and neurocognitive outcomes. This imbalance reflects the current state of the literature rather than a conceptual oversight. Indeed, while numerous wearable-based protocols have been developed to enhance physical activity and fitness, these studies typically assess outcomes limited to physiological parameters, physical performance, or health risk reduction, with little attention to concomitant changes in psychological balance or cognitive functioning. In contrast, the triadic model proposed here aims to provide a broader conceptual lens through which well-being can be understood as the result of synergistic interactions among cognitive, emotional, and physical domains. The present review, therefore, serves as a first step toward mapping the existing evidence, while underscoring the need for future research to adopt integrative designs that systematically include physical fitness indices alongside psychological and neurocognitive measures.

### Conclusive Theoretical–Practical Notes

A key conceptual contribution of this review is its reframing of intervention logic: from treatment to training, from passive reception to active participation. While conventional healthcare models often consider the individual as a recipient of expert-driven treatment—where success hinges on adherence to prescribed procedures—neuroempowerment introduces a paradigm of active self-regulation and personal agency. In this perspective, the participant is no longer a passive subject to be corrected, but an engaged agent whose motivational, attentional, and self-reflective capacities play a constitutive role in the effectiveness of the intervention [69,70,71,72].

This shift has both theoretical and practical implications. Theoretically, it suggests that well-being cannot be externally imposed; it must be cultivated through participatory processes that enhance awareness, foster self-efficacy, and support volitional engagement. Practically, it positions engagement and agency as modifiable levers of intervention efficacy. Protocols that fail to consider these elements risk underestimating the complexity of change processes and over-relying on prescriptive models.

Indeed, multiple studies in the reviewed literature demonstrate that engagement is not merely a prerequisite for adherence but a driver of neuroplastic change. The act of training—through repeated interaction with mindful attention, bodily awareness, or neuro/biofeedback—mobilizes cognitive resources, emotional regulation strategies, and motivational systems that support long-term adaptation. This is especially evident in protocols involving applied psychophysiology devices, where the individual must actively learn to recognize, interpret, and modulate internal states based on real-time physiological information [8,23,24,36,39,53,58,61].

We propose that, in this context, neurotechnologies serve a dual function. On one hand, they operate as precision tools for capturing subtle shifts in neurophysiological functioning. On the other hand, they act as relational and educational mediators, scaffolding the user’s learning process by transforming implicit, pre-reflective experiences into explicit, self-directed insights. This ‘technologically mediated embodiment’ enables users to build novel forms of self-awareness, grounded in lived physiological states, and promotes adaptive self-regulation.

Moreover, wearable devices enhance ecological validity by extending intervention contexts beyond clinical or laboratory settings into the flow of daily life [25,73,74,75,76,77,78]. In doing so, they may democratise access to training, support self-paced learning, and reduce structural barriers to participation—thereby enhancing both scalability and inclusivity. Importantly, they also allow for dynamic personalization, as real-time data can be used to tailor feedback and adapt difficulty levels according to individual progress and responsiveness.

To fully capitalize on this potential, the field could embrace an integrated approach to assessment. Relying uniquely on self-report measures—though still valuable for capturing subjective experience—is insufficient to map the multidimensional effects of empowerment protocols. Instead, a triangulated framework is needed: one that integrates neurocognitive assessments, psychophysiological metrics, and phenomenological self-report tools. Such multimodal approaches allow for a more sensitive and ecologically valid understanding of change processes, and are particularly useful in capturing the interplay between objective function and subjective awareness.

Furthermore, future research should include design features that explicitly sustain and measure engagement over time. This includes the use of interactive interfaces, adaptive goal-setting, feedback customization, and gamification elements that do not trivialize but rather enhance the meaning of participation. The long-term success of empowerment-based interventions depends not only on the strength of their immediate effects but also on their ability to maintain motivational resonance and experiential relevance for participants.

Several methodological limitations in the reviewed studies—such as small sample sizes, short durations, lack of active control groups, and poor integration of objective measures—highlight the need for more rigorous and theoretically grounded research designs. Future studies should keep adopting longitudinal frameworks with pre-, post-, and follow-up assessments that reflect the temporal dynamics of learning and adaptation. Additionally, participatory design approaches—where users contribute to the co-construction of protocols—may enhance both adherence and outcome relevance.

Finally, this review supports the development of a next-generation intervention model that we might call ‘adaptive neuroempowerment’. Such a model would synthesize insights from neuroscience, embodied cognition, and positive psychology, while harnessing the capacities of wearable technologies to create customizable, self-directed, and relationally meaningful interventions. Rather than prescribing well-being, these protocols aim to cultivate the agentive capacities through which well-being is enacted, learned, and sustained over time.

In conclusion, remote neuroempowerment protocols might represent a promising frontier for promoting health and well-being in the general population. Their success, however, rests not only on their scientific sophistication but also on their potential for fostering engagement and commitment, supporting agency, and empowering individuals as active protagonists in their own cognitive, emotional, and physiological development.

## Figures and Tables

**Table 1:** Synopsis of studies included in the scoping review, grouped by research design features (control conditions; number of assessment sessions).

Study	Sample	Method	Intervention	Assessment	Outcome Measurements	Duration	Component of Triadic Model
Balconi et al., 2019 [38]	Young adults and adults N = 50 Age: Mean = 24.20; SD = 6.99	Longitudinal (2 assessment sessions) Control condition: active	At-home mindfulness-based training Wearable neurofeedback	Autonomic markers + Cognitive task + Self-report questionnaires + Driving behaviour	Resting-state autonomic markers (HR, HRV); Task-related autonomic markers (HR, HRV) Attentional Matrices; Multiple Features Targets Cancellation (MFTC); Stroop test; Stroop-like task Five Facet Mindfulness Questionnaire (FFMQ) Driving Behavior Questionnaire (DBQ); Index of Driving Performance (based on travel distance, time slots, real-time respect for speed limits, real-time accelerations and decelerations)	3 weeks	PB + NE
Bhayee et al., 2016 [23]	Adults N = 26 Age: Mean = 32.65; SD = 4.75	Longitudinal (2 assessment sessions) Control condition: active	App-based mindfulness training Wearable neurofeedback	Cognitive tasks + self-report questionnaires	Stroop test; d2 task; Digit Span tasks Brief Symptom Inventory (BSI); Freiburg Mindfulness Inventory (FMI); Positive and Negative Affective Schedule (PANAS); World Health Organization Quality of Life scale brief version (WHOQOL-BREF); Big Five Inventory (BFI)	6 weeks	PB + NE
Crivelli et al., 2019 [24]	Young adults N = 40 Age: Mean = 23.47; SD = 2.33	Longitudinal (2 assessment sessions) Control condition: active	At-home mindfulness-based training Wearable neurofeedback	EEG + Cognitive task	Resting-state EEG (alpha–beta ratio; alpha blocking index); Task-related EEG (N2 event-related potential, amplitude, and latency) MIDA battery; Stroop task	4 weeks	NE
Crivelli et al., 2019 [39]	Young adults and athletes N = 50 Age: Mean = 22.94; SD = 2.22	Longitudinal (2 assessment sessions) Control condition: active Other: healthy participants vs. athletes	At-home mindfulness-based training Wearable neurofeedback	EEG + Cognitive task + Self-report questionnaires	Task-related EEG (N2 event-related potential, amplitude, and latency) MIDA battery; Stroop task Perceived Stress Scale (PSS); State/Trait Anxiety Inventory (STAI); Five-Facet Mindfulness Questionnaire (FFMQ)	2 weeks	PB + NE
Karing, 2023 [40]	Young adults and adults N = 50 Age: Mean = 24.12; SD = 6.23	Longitudinal (2 assessment sessions) Control condition: active	Online mindfulness program No wearable devices	Self-report questionnaires	Five-Facet Mindfulness Questionnaire (FFMQ); Patient Health Questionnaire-8 scale (PHQ-8); 7-item Generalized Anxiety Disorder scale (GAD-7); General Life Satisfaction Scale	7 weeks	PB
Kay & Young, 2022 [41]	Young adults N = 201 Age: Mean = 24.60; SD = 2.90	Longitudinal (2 assessment sessions) Control condition: active	At-home mindfulness, yoga, and mental training program No wearable devices	Self-report questionnaires	Five-Facet Mindfulness Questionnaire (FFMQ); Conscientiousness subscale of the HEXACO short form; Authenticity Scale; Psychological Well-Being Scale (PWB)	8 weeks	PB
Noone & Hogan, 2018 [42]	Young adults N = 71 Age: Mean = 20.56; SD = 3.52	Longitudinal (2 assessment sessions) Control condition: active	App-based mindfulness training No wearable devices	Cognitive tasks + Self-report questionnaires	Sternberg Working Memory Task; Halpern Critical Thinking Assessment (HCTA); Heuristics and Biases Five Facet Mindfulness Questionnaire (FFMQ); Positive and Negative Affect Schedule (PANAS); Warwick-Edinburgh Mental Well-Being Scale; Real-world outcomes inventory	6 weeks	PB + NE
Autenrieth et al., 2023 [43]	Young adults N = 17 Age: Mean = 23.56; SD = 2.26	Longitudinal (3 assessment sessions) Control condition: active	At-home neuroempowerment training Wearable neurofeedback	EEG + Self-report questionnaire	Task-related EEG (log-transformed SMR, theta, and beta power) Ad hoc questionnaire on subjective training experience; Open question on mental strategies	2 weeks	NE
Coelhoso et al., 2019 [44]	Adult female professionals N = 305 Age: Mean = 34.58; SD = 7.63	Longitudinal (3 assessment sessions) Control condition: active	App-based meditation and positive psychology training No wearable devices	Self-report questionnaires	Perceived Stress Scale (PSS); World Health Organization-Five Well-Being Index (WHO-5); Ad hoc questionnaire on Subjective Symptoms of Stress and Well-Being	8 weeks	PB
Khatib et al., 2022 [45]	Adult professionals N = 50 Age: Median = 44; SD = n/a	Longitudinal (3 assessment sessions) Control condition: active	Online mindfulness program No wearable devices	Self-report questionnaires	Aggression Questionnaire (AGQ); Perceived Stress Scale (PSS); Beck Depression Inventory-II (BDI-II); Difficulties in Emotion Regulation Scale (DERS); State Anxiety Inventory (SAI); Five Facet Mindfulness Questionnaire (FFMQ)	8 weeks	PB
Mak et al., 2018 [46]	Adults N = 2161 Age: Mean = 33.64; SD = 12.06	Longitudinal (3 assessment sessions) Control condition: active	App-based mindfulness training No wearable devices	Self-report questionnaires	World Health Organization-Five Well-Being Index (WHO-5); 6-item Kessler Psychological Distress Scale (K6); Mindful Attention and Awareness Scale (MAAS); Self-Compassion Scale; Depressed Mood and Anxiety subscales of the Affective Control Scale; Discomfort with Ambiguity subscale of the Need for Closure Scale	4 weeks	PB
Smith et al., 2024 [47]	Young adults N = 326 Age: Mean = 23.61; SD = 5.39	Longitudinal (3 assessment sessions) Control condition: active	Online self-awareness and affective empowerment training No wearable devices	Socio-affective tasks + Self-report questionnaires	Perception of Affect Task (PAT); Poor Skills subscale of the Managing Emotions of Others Scale (MEOS); brief Situational Test of Emotion Management (STEM-B) Levels of Emotional Awareness Scale (LEAS); Multidimensional Assessment of Interoceptive Awareness (MAIA); Five-Facet Mindfulness Questionnaire (FFMQ; Multidimensional Experiential Avoidance Questionnaire (MEAQ); Emotional Regulation Questionnaire (ERQ); Difficulties in Emotion Regulation Scale (DERS)	1/3 weeks	PB + NE
Brandão et al., 2024 [48]	Young adults N = 106 Age: Mean = 21.04; SD = 4.59	Longitudinal (3 assessment sessions) Control condition: both active and passive	Online mindfulness, yoga, and mental training program No wearable devices	Self-report questionnaires	Depression Anxiety Stress Scale-21 (DASS–21); Self-Compassion Scale; Self-Concept Clinical Inventory; Emotion Regulation of Others and Self (EROS); Spiritual Well-Being Questionnaire; Subjective Happiness Scale	6 weeks	PB
Baminiwatta et al., 2024 [49]	Adult professionals N = 80 Age: Mean = 39.80; SD = 9.66	Longitudinal (2 assessment sessions) Control condition: passive	Online mindfulness program No wearable devices	Self-report questionnaires	Perceived Stress Scale (PSS); World Health Organization-Five Well-Being Index (WHO-5)	6 weeks	PB
Bossi et al., 2022 [50]	Adult professionals N = 132 Age: Mean = 41.63; SD = 10.13	Longitudinal (2 assessment sessions) Control condition: passive	Online mindfulness program No wearable devices	Self-report questionnaires	Ad hoc questionnaire on the use of habits as coping strategies; Five Facet Mindfulness Questionnaire short form (sh-FFMQ); Emotion Regulation Questionnaire (ERQ); Positive and Negative Affect Scale (PANAS); Depression Anxiety Stress Scale-21 (DASS-21); Resilience Scale for Adults (RSA); Insomnia Severity Index (ISI)	8 weeks	PB
Kappen et al., 2019 [51]	Adults in a romantic relationship N = 113 Age: Mean = 27.27; SD = 8.31	Longitudinal (2 assessment sessions) Control condition: passive	Online mindfulness program No wearable devices	Self-report questionnaires	Partner Acceptance Scale (PAS); Five Facet Mindfulness Questionnaire short form (sh-FFMQ); Global Satisfaction subscale from the Investment Model Scale	2 weeks	PB
Nadler et al., 2020 [52]	Adult professionals N = 102 Age: Mean = 48.31; SD = 10.35	Longitudinal (2 assessment sessions) Control condition: passive	Online mindfulness program No wearable devices	Self-report questionnaires	Five-Facet Mindfulness Questionnaire (FFMQ); Perceived Stress Scale (PSS); Brief Resilience Scale (BRS); Positive and Negative Affect Schedule (PANAS); Multidimensional Emotional Intelligence Assessment—Workplace (MEIA-W); Workplace Competency Assessment	8 weeks	PB
Pal et al., 2022 [53]	Young adults N = 66 Age: Mean = n/a; SD = n/a	Longitudinal (2 assessment sessions) Control condition: passive	At-home mindfulness and yoga training program Wearable ECG band	Autonomic markers + Self-report questionnaire	Resting-state autonomic markers (HRV) Self-Rating Anxiety Scale (SAS)	6 weeks	PB
Spelt et al., 2019 [54]	Adults of low socioeconomic status N = 195 Age: Mean = 41.07; SD = 12.53	Longitudinal (2 assessment sessions) Control condition: passive	App-based lifestyle coaching Activity tracker	Self-report questionnaires	International Physical Activity Questionnaire short version (S-IPAQ); Warwick–Edinburgh Mental Well-Being Scale (WEMWBS); ad hoc questionnaire on underlying motivations of behaviour	19 weeks	PB + PF
Bostock et al., 2019 [55]	Adult professionals N = 238 Age: Mean = 35.50; SD = 7.70	Longitudinal (3 assessment sessions) Control condition: passive	App-based mindfulness training No wearable devices	Autonomic markers + Self-report questionnaires	Resting-state autonomic markers (blood pressure measurement) Warwick Edinburgh Mental Well-Being Scale (WEMWBS); Hospital Anxiety and Depression Scale (HADS); Job Demands and Job Control subscales of the Whitehall II study questionnaire; Ad hoc questionnaire on social support at work; 7 items from the Freiburg Mindfulness Inventory	8 weeks	PB
Champion et al., 2018 [56]	Adults N = 74 Age: Mean = 39.40; SD = 5.76	Longitudinal (3 assessment sessions) Control condition: passive	App-based mindfulness training No wearable devices	Self-report questionnaires	General Health Questionnaire 28 (GHQ); Satisfaction with Life Scale (SWLS); Perceived Stress Scale (PSS); Wagnild Resilience Scale (WRS); ad hoc questionnaire on engagement and experience	4/5 weeks	PB
Ivtzan et al., 2016 [57]	Adults, professionals, and meditators N = 168 Age: Mean = 40.86; SD = 11.31	Longitudinal (3 assessment sessions) Control condition: passive	Online mindfulness program No wearable devices	Self-report questionnaires	Pemberton Happiness Index (PHI); Perceived stress scale (PSS); Beck’s depression inventory-II (BDI-II); Freiburg Mindfulness Inventory (FMI); 6-item Gratitude Questionnaire (GQ6); Self-Compassion Scale short form (sh-SCS); Autonomy subscale of the Psychological Well-Being scale (PWB); Generalised Self-Efficacy scale (GSE); Presence subscale of the Meaning in Life Questionnaire (MLQ); Compassion for Others Scale (COS); Present Moment subscale of the Appreciation Inventory scale (AI)	8 weeks	PB
Min et al., 2023 [58]	Adults professionals N = 93 Age: Mean = 38.64; SD = 10.87	Longitudinal (3 assessment sessions) Control condition: passive	At-home mindfulness-based training Wearable neurofeedback	EEG + Autonomic markers + Self-report questionnaires	Resting-state EEG (standard EEG bands power; alpha-beta ratio; beta-theta ratio) Resting-state autonomic markers (HRV) Perceived Stress Scale (PSS); Brief Resilience Scale (BRS); Mindfulness Attention Awareness Scale (MAAS); Korean Emotional Labor Scale (KELS); Korean Standard Occupational Stress Scale short form (KOSS); Athens Insomnia Scale (AIS); 9-item Patient Health Questionnaire (PHQ-9)	4 weeks	PB
Mota et al., 2023 [59]	Young adults N = 97 Age: Mean = 18.14; SD = 1.79	Longitudinal (3 assessment sessions) Control condition: passive	Online lifestyle coaching No wearable devices	Self-report questionnaires + Dietary data + Anthropometric measurements	Stress Indicator Questionnaire Dietary intake; Nutrient Rich Food (NRF) index Body composition indices; Body Mass Index (BMI)	12 weeks	PB + PF
Querstret et al., 2018 [60]	Adults N = 118 Age: Mean = 40.68; SD = 10.45	Longitudinal (4 assessment sessions) Control condition: passive	At-home mindfulness-based training No wearable devices	Self-report questionnaires	Perceived Stress Scale (PSS); 9-item Patient Health Questionnaire (PHQ-9); 7-item Generalized Anxiety Disorder (GAD-7); Five Facet Mindfulness Questionnaire short form (sh-FFMQ)	4 weeks	PB
Crivelli et al., 2019 [61]	Senior managers N = 16 Age: Mean = 44.38; SD = 6.22	Longitudinal (2 assessment sessions) Control condition: absent	At-home mindfulness-based training Wearable neurofeedback	EEG + Autonomic markers + Cognitive task + Self-report questionnaires	Resting-state EEG (alpha–beta ratio; alpha blocking index); Task-related EEG (N2 event-related potential, amplitude, and latency) Resting-state and task-related autonomic markers (HR; HRV) MIDA battery; Stroop task Perceived Stress Scale (PSS); State/Trait Anxiety Inventory (STAI); Profile of Mood States (POMS)	2 weeks	PB + NE
Moscaleski et al., 2022 [62]	Female athletes N = 9 Age: Mean = 25.70; SD = 7.00	Longitudinal (2 assessment sessions) Control condition: absent	At-home physical and mental training No wearable devices	Self-report questionnaires	Internal Training Load (ITL); Session Rating of Perceived Exertion (s-RPE); ad hoc scale on training motivation; Well-being questionnaire	16 weeks	PB + PF
Kolken et al., 2023 [63]	Adults with sleep difficulties N = 37 Age: Mean = 48.20; SD = n/a	Longitudinal (2 assessment sessions) Control condition: absent Other: neurofeedback learners vs. non-learners	At-home neuroempowerment training Wearable neurofeedback	Cognitive tasks	Continuous Performance/Working Memory task (CPT/WM); Stroop task; Trail Making test; Forward and Backward Digit Span test; Tapping test; Corsi Blocks test	8/12 weeks	NE

Note: PB: psychological balance; NE: neurocognitive efficiency; PF: physical fitness.

## Data Availability

No new data were created or analysed in this study.
